# Gastrointestinal *Talaromyces marneffei* infection in a man with AIDS: A case report

**DOI:** 10.1097/MD.0000000000033424

**Published:** 2022-04-07

**Authors:** Renping Peng, Zhipeng Peng, Minhong Mou, Qiudong Wang, Man Huang, Jun Zou

**Affiliations:** a Department of Endoscopy, The Fourth People’s Hospital of Nanning, Nanning, China; b Department of Dermatology, The Fourth People’s Hospital of Nanning, Nanning, China; c Department of Pathology, The Fourth People’s Hospital of Nanning, Nanning, China; d Department of Laboratory of Infectious Diseases, The Fourth People’s Hospital of Nanning, Nanning, China; e Department of Infectious Disease, The Fourth People’s Hospital of Nanning, Nanning, China.

**Keywords:** acquired immunodeficiency syndrome, case report, *g*astrointestinal tract, *Talaromyces marneffei* infection

## Abstract

Reports of intestinal *Talaromyces marneffei* infection have increased year by year, but those of gastric infection remain rare. Here, we report disseminated talaromycosis with gastric and intestinal ulcers in an AIDS patient who was treated by antifungal agents and a proton pump inhibitor and achieved a satisfactory outcome.

**Patient concerns:**

A 49-year-old man developed a gastrointestinal illness with main abdominal distension, poor appetite and a positive HIV infection to our AIDS clinical treatment center.

**Diagnoses:**

Electronic gastrointestinal endoscopy showed that the patient had multiple ulcers in the gastric angle, gastric antrum and large intestine. Gastric *Helicobacter pylori* infection was ruled out by paraulcerative histopathological analysis and a C14 urea breath test. The diagnosis was confirmed by gastroenteroscopic biopsy and metagenomic next-generation sequencing of gastric ulcer tissue.

**Interventions:**

Symptomatic and supportive treatments [a proton pump inhibitor and gastrointestinal motility promotion] were initiated. The patient was prescribed sequential antifungal therapy with amphotericin B (0.5 mg/kg·d, 2 weeks) and itraconazole (200 mg, q12h, 10 weeks), and then followed with itraconazole for long-term secondary prevention (200 mg, qd).

**Outcomes:**

The combined use of antifungal agents and a proton pump inhibitor improved the patient’s condition, and he was discharged home 20 days later. He had no gastrointestinal symptom during 1 year of telephone-based follow-up.

**Lessons:**

In endemic areas, clinicians should be alert to the possibility of *Talaromyces marneffei* infection presenting with gastric ulcers in patients with AIDS, after excluding *Helicobacter pylori* infection.

## 1. Introduction

*Talaromyces marneffei* (TM; previously known as *Penicillium marneffei*), a temperature-dependent dimorphic fungus, has emerged as a significant pathogen causing potentially fatal systemic mycosis in patients with advanced HIV infection and other immunosuppressive conditions. It is endemic in areas north of the equator to 25° to 30° north latitude, such as northern Thailand, Malaysia, Vietnam, northern India, southern China, Hong Kong, and Taiwan, and the epidemic area is expanding gradually with the year-by-year increase in the floating population.^[1]^ TM is transmitted to the whole body via the blood, lymphatic system (including the skin), or respiratory, digestive, or reticuloendothelial system.^[2]^ About 1.9% of talaromycosis cases are enteric infections.^[3]^ In recent years, reports of intestinal TM infection have increased.^[4–7]^ Those of stomach infection remain rare.

Here, we report a case of gastrointestinal ulcers in a patient with AIDS infected with TM. Histopathological analysis and metagenomic next-generation sequencing (mNGS) of the ulcer tissue confirmed that the pathogen was TM.

## 2. Case report

A 49-year-old man was admitted with a gastrointestinal illness to the Fourth People’s Hospital of Nanning, China. He presented with abdominal distension, poor appetite, occasional belching and acid reflux, and 7.5 kg weight loss in 1 month; he had no fever, cough, abdominal pain, or diarrhea. The patient was HIV positive but was not on antiretroviral therapy. Physical examination revealed a rash on the face, but no oral mucosal involvement. The patient had no hepatomegaly or splenomegaly. Blood tests revealed anemia (hemoglobin concentration 80 g/L, red blood cell count 3.07 × 10^12^/L). The CD4^+^ and CD8^+^ T-lymphocyte counts were 7 and 35 cells/µL, respectively. Biochemical analysis showed that the patient’s aspartate aminotransferase level was 117 U/L, his alanine aminotransferase level was 82 U/L, and his 1-3-β-D-glucan level was 838.1 pg/mL. Results of a *Cryptococcus neoformans* capsular polysaccharide antigen test, a *Mycobacterium tuberculosis* – associated gamma interferon spot test, and blood and urine cytomegalovirus load tests were negative. A fecal occult blood test yielded positive results and a blood culture showed TM growth.

**Figure 1.**
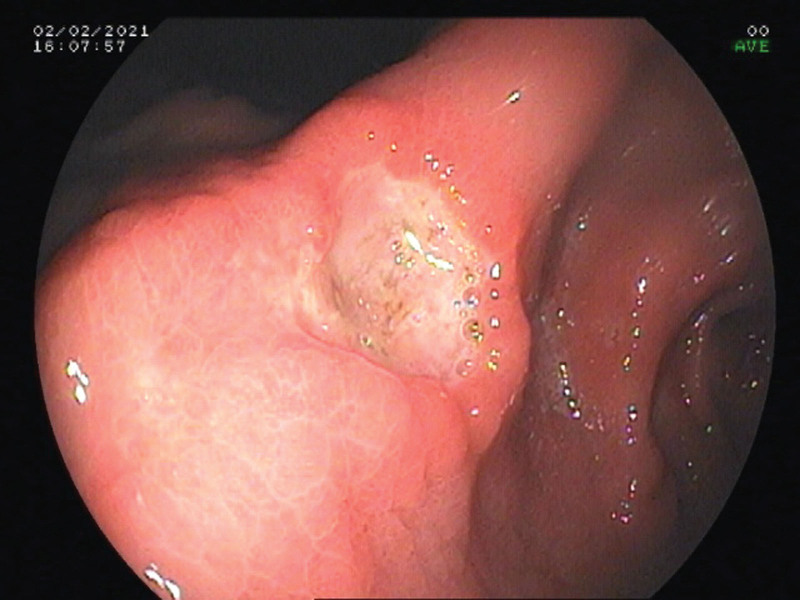
Gastroscopic view of a deep ulcer in the gastric angle.

**Figure 2.**
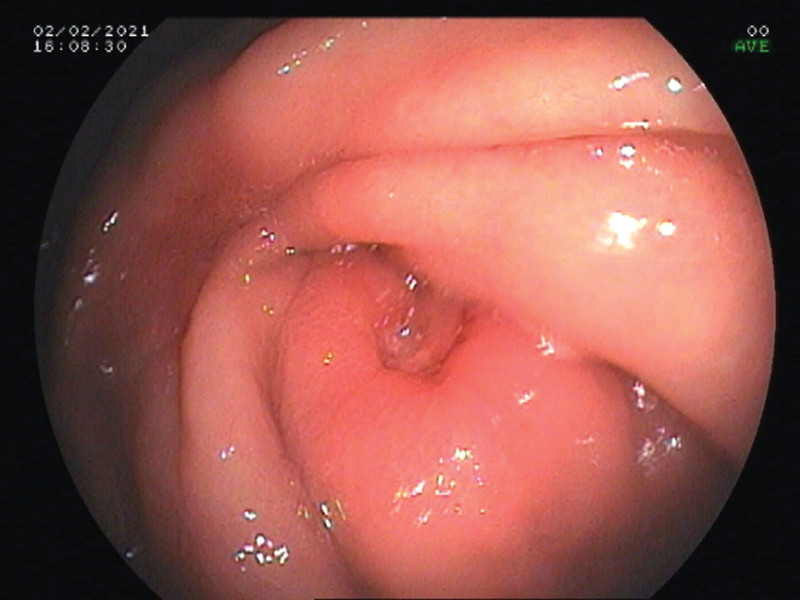
Gastroscopic view of an ulcer in the gastric antrum.

**Figure 3.**
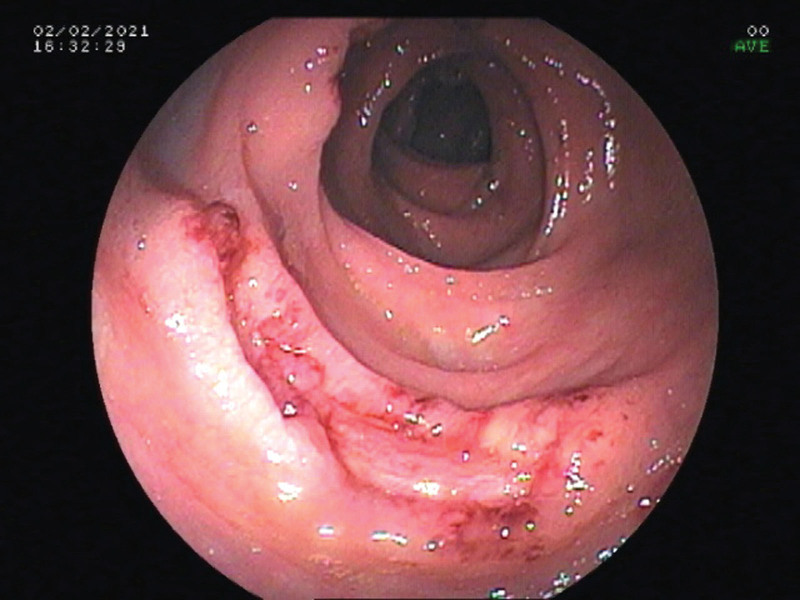
Colonoscopic view of a deep ulcer in the colon.

**Figure 4.**
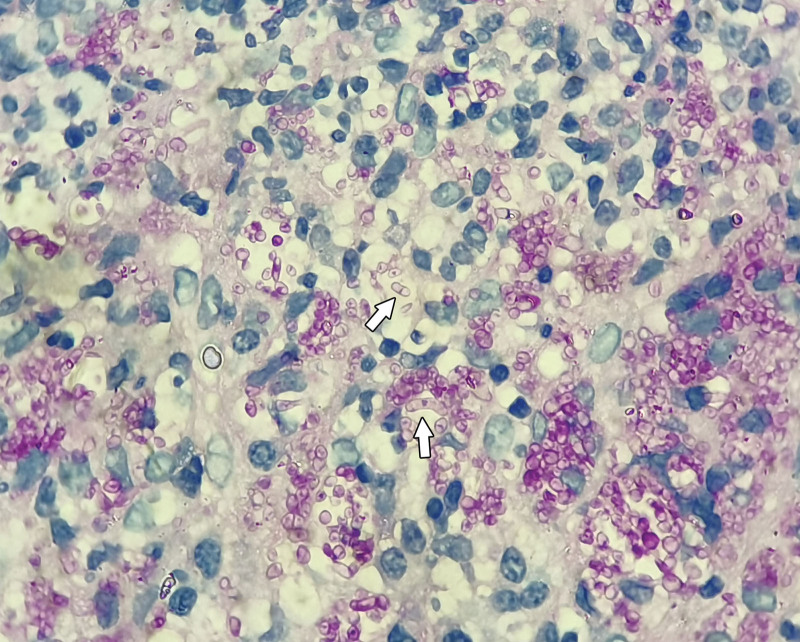
Periodic acid–Schiff staining with diastase (×1000) of a pathological section showing numerous round and oval spores with a transverse septum.

Electronic gastrointestinal endoscopy showed that the patient had multiple ulcers in the gastric angle (Fig. 1), gastric antrum (Fig. 2), and large intestine (Fig. 3). The mucosa surrounding the ulcers was swollen, suggesting hyperplastic or congestive edema. A 14C urea breath test was negative for *Helicobacter pylori* (HP). Biopsies of ulcerated gastrointestinal tissue were performed, and the specimens showed abundant purple-red sausage-shaped fungi with septa on periodic acid–Schiff staining with diastase (Fig. 4); adjacent gastric ulcer tissue was HP negative. mNGS of the gastric ulcer tissue confirmed TM infection.

Symptomatic and supportive treatments [a proton pump inhibitor and gastrointestinal motility promotion] were initiated. The patient was prescribed sequential antifungal therapy with amphotericin B (0.5 mg/kg·d, 2 weeks) and itraconazole (200 mg, q12h, 10 weeks). These treatments relieved his condition, and he was discharged after being hospitalized for 20 days. Because the patient requested to return to the local hospital for antiretroviral treatment and follow up, gastroenterological endoscopy was not repeated. After 1 year of telephone-based follow-up, the patient had no gastrointestinal symptom. His CD4^+^ T-lymphocyte count, determined at the local hospital, was 89 cells/μL.

## 3. Discussion

TM infection is a common fungal disease among HIV-infected patients living in or having visited endemic areas. A report on gastrointestinal talaromycosis, an uncommon form of the infection, in 19 patients indicated that the most common clinical features were fever, abdominal pain, diarrhea, bloody stools, and weight loss.^[5]^ Similarly, the clinical presentation of intestinal TM infection in 31 HIV-infected patients included fever (38.71%), abdominal pain (38.71%), diarrhea (25.81%), and CD4^+^ T-lymphocyte count < 50 cells/µL (90.32%).^[8]^ A patient with gastric mucosal erosions and intestinal ulcers combined with TM infection presented with diarrhea, weight loss, fever, and a CD4^+^ T-lymphocyte count of 11 cells/µL.^[9]^ Our patient’s presentation was somewhat different, as he had a low CD4^+^ T-lymphocyte count and weight loss but no abdominal pain, fever, or diarrhea; he did have abdominal distension, poor appetite, and skin lesions. Atypical gastrointestinal symptoms may lead clinicians to misdiagnose TM infection as a common digestive tract disease, leading to delays in treatment.

TM infection in the digestive tract almost always involves the intestinal tract, with few cases involving the stomach. Erosions and ulcers are the most common intestinal lesions of TM infection.^[5,8]^ The present case is unusual in that the patient had gastric and intestinal ulcers. The diagnosis of TM infection was confirmed and HP infection was excluded by blood culture, a C14 breath test, and pathological analysis and mNGS of biopsy specimens. To our knowledge, this case in a patient with AIDS is the first in which TM was isolated from gastric ulcer tissue, confirming that the manifestations of TM infection are not limited to intestinal ulcers.

The early administration of the antifungal agents amphotericin B and itraconazole effectively treats talaromycosis and improves patient survival.^[10]^ Antifungal and proton pump inhibitor therapy significantly relieved the clinical symptoms of the patient described here, who remained relapse free at 1 year. If talaromycosis is not diagnosed in a timely manner and treated appropriately, its mortality rate can reach 50.6%,^[11]^ and even up to 91%.^[12]^ As cultures from the marrow, blood, and feces of infected individuals are not always TM positive and the clinical manifestations of TM infection in the digestive tract are not specific, the disease could be misdiagnosed or overlooked. In TM endemic areas, endoscopy should be performed to look for gastric TM infection in HIV-infected patients presenting with atypical gastrointestinal symptoms and CD4^+^ T-lymphocyte counts < 50 cells/µL.

Disseminated talaromycosis with the simultaneous presence of gastric and intestinal ulcers caused by TM is rare. In the case described here, it was characterized mainly by abdominal distension and poor appetite, with no fever, abdominal pain, or diarrhea. In TM endemic areas, clinicians should be alert to the possibility of TM infection presenting with gastric ulcers in patients with AIDS, after excluding HP infection. The histopathological analysis and mNGS of gastrointestinal samples can confirm the identification of TM infection in the stomach and intestine.

## Acknowledgments

We thank Medjaden Inc. for scientific editing of this manuscript.

## Author contributions

**Conceptualization:** Jun Zou.

Funding acquisition: Jun Zou.

Investigation: Renping Peng, Zhipeng Peng.

Methodology: Jun Zou.

Supervision: Jun Zou.

Visualization: Renping Peng, Minhong Mou, Qiudong Wang, Man Huang.

Writing – original draft: Renping Peng, Zhipeng Peng.

Writing – review & editing: Jun Zou.

## References

[1] Cao C Xi L Chaturvedi V. Talaromycosis (Penicilliosis) Due to Talaromyces (Penicillium) marneffei: insights into the clinical trends of a major fungal disease 60 years after the discovery of the pathogen. Mycopathologia. 2019;184:709–20. 31811603 10.1007/s11046-019-00410-2

[2] Qiu Y Zhang J Liu G. Retrospective analysis of 14 cases of disseminated Penicillium marneffei infection with osteolytic lesions. BMC Infect Dis. 2015;15:47. 25656710 10.1186/s12879-015-0782-6 PMC4322545

[3] Zhou Y Liu Y Wen Y. Gastrointestinal manifestations of *Talaromyces marneffei* infection in an HIV-infected patient rapidly verified by metagenomic next-generation sequencing: a case report. BMC Infect Dis. 2021;21:376. 33882850 10.1186/s12879-021-06063-1 PMC8059157

[4] Philip Sridhar R Coelho VV Roopavathana B. Opportunistic penicilliosis infection causing intestinal obstruction in people living with HIV complicating antiretroviral therapy. BMJ Case Rep. 2020;13:e230121. 10.1136/bcr-2019-230121 PMC7046417 32060105

[5] Pan M Huang J Qiu Y. Assessment of *Talaromyces marneffei* infection of the intestine in three patients and a systematic review of case reports. Open Forum Infect Dis. 2020;7:ofaa128. 32523970 10.1093/ofid/ofaa128 PMC7264840

[6] Zhao YK Liu JY Liu JH. Recurrent *Talaromyces marneffei* infection presenting with intestinal obstruction in a patient with systemic lupus erythematosus. Mycopathologia. 2020;185:717–26. 32647906 10.1007/s11046-020-00469-2

[7] Cui X Su F Ye H. Disseminated talaromycosis complicated by recurrent gastrointestinal bleeding and hemorrhagic shock: a case report. BMC Infect Dis. 2022;22:238. 35264100 10.1186/s12879-022-07230-8 PMC8905750

[8] Xie Z Lai J Peng R. Clinical characteristics of HIV-associated *Talaromyces marneffei* infection of intestine in Southern China. Int J Infect Dis. 2022;120:48–50. 35398298 10.1016/j.ijid.2022.03.057

[9] Hung HG Lok KH. Intestinal Penicillium marneffei: an unusual cause of chronic diarrhea in an AIDS patient. J Dig Dis. 2010;11:189–91. 20579223 10.1111/j.1751-2980.2010.00435.x

[10] Le T Kinh NV Cuc NTK.; IVAP Investigators. A Trial of Itraconazole or Amphotericin B for HIV-associated talaromycosis. N Engl J Med. 2017;376:2329–40. 28614691 10.1056/NEJMoa1613306

[11] Hu Y Zhang J Li X. Penicillium marneffei infection: an emerging disease in mainland China. Mycopathologia. 2013;175:57–67. 22983901 10.1007/s11046-012-9577-0

[12] Liu B Ping FU. Research progress of Penicilliosis Marneffei. J Dermatol Venereol. 2010;32:26–8.

